# Pretreatment of Grape Stalks by Fungi: Effect on Bioactive Compounds, Fiber Composition, Saccharification Kinetics and Monosaccharides Ratio

**DOI:** 10.3390/ijerph17165900

**Published:** 2020-08-14

**Authors:** Joana M.C. Fernandes, Irene Fraga, Rose M.O.F. Sousa, Miguel A.M. Rodrigues, Ana Sampaio, Rui M.F. Bezerra, Albino A. Dias

**Affiliations:** CITAB-Centre for the Research and Technology of Agro-Environmental and Biological Sciences, UTAD—Universidade de Trás-os-Montes e Alto Douro, 5000-801 Vila Real, Portugal; joanaf@utad.pt (J.M.C.F.); ifraga@utad.pt (I.F.); rosesousa@utad.pt (R.M.O.F.S.); mrodrigu@utad.pt (M.A.M.R.); asampaio@utad.pt (A.S.); bezerra@utad.pt (R.M.F.B.)

**Keywords:** antioxidants, biorefinery, fungal pretreatment, grape stalks, saccharification, waste valorization

## Abstract

Grape stalks, an inedible lignocellulosic residue from winemaking and agro-industrial grape juice production, can be valorized as a source of bioactive compounds and as feedstock for the saccharification and bioconversion of soluble sugars. Solid-state fermentation (SSF) by six white-rot fungi was applied as pretreatment. Fiber composition, free radical scavenging activity, four ligninolytic, and three hydrolytic enzyme activities were determined. Saccharification kinetics, yield, and productivity were evaluated and complemented with scanning electron microscopy (SEM), high performance liquid chromatography (HPLC) quantification of monosaccharides, and principal component analysis (PCA). After SSF, the biomass exhibited a drastic free radical scavenging activity decrease and the main enzymes produced were manganese-dependent peroxidase and xylanase. Scanning electron microscopy revealed the erosion of cell walls, and PCA exhibited a negative correlation between saccharification, and neutral detergent fiber and acid detergent lignin. *Phlebia rufa* pretreated biomass gave the highest sugars yield and productivity, representing a nearly three-fold increase compared to untreated samples. Also, monosaccharides quantification revealed that the 1:1 ratio of glucose to the sum of xylose plus galactose changes to the value of 2:1 after pretreatment. In this work, and for the first time, *P. rufa* proved to be an effective pretreatment of grape stalks for the saccharification and further bioconversion into value-added chemicals. In addition, lignocellulolytic enzymes were also produced through SSF.

## 1. Introduction

The human dependence on non-renewable resources, fossil fuels depletion, and climate issues [1] have influenced an increasing trend in the adoption of decarbonization policies [2], mainly in critical areas such as the transport sector, which accounts for about 23% of total CO_2_ emissions [3]. In this context, the European Union is committed to a 25% increase in biofuels production for the transport sector by 2030 in order to reach a substantial reduction in greenhouse gas emissions [4]. Biofuels come from carbon-neutral sources, such as different types of vegetable biomass, whose major macromolecules are cellulose, hemicellulose, starch, and lignin. Concerning lignocellulosic biomass, the content of cellulose, hemicellulose, and lignin may range between 30–50%, 20–40%, and 10–30%, respectively [5].

Grape stalks (*Vitis vinifera* L.), an inedible lignocellulosic by-product, are annually obtained from destemming operations carried out at wineries, prior to the vinification process, and at agro-industrial facilities of grape juice production (between middle August to October in Mediterranean countries). Currently, the most common destinations for grape stalks are lower value-added options such as landfilling, landfarming (spreading and incorporation into agricultural soils), or composting. However, as previously reported by Nicolini et al. [6], grape stalks digestibility increases after fungal fermentation. Grape stalks were also used as a cheap carbon source and an inducer of ligninolytic enzymes production for application in bioremediation processes, such as the removal of textile dyes from effluents [7,8]. Another alternative that will generate greater economic benefits is the extraction of bioactive compounds followed by the saccharification of structural polysaccharides, i.e., applying the biorefinery concept. Further, hydrolyzed biomass can be converted into carbon-neutral high value-added products [9]. However, due to the composite structure and recalcitrance of the lignocellulosic matrix, effective accessibility to structural polysaccharides is the largest challenge [10] for the emergence of a sustainable development based on a circular economy. Accessibility increase to carbohydrates represents one of the most expensive steps in the process of bioethanol production with a contribution of about 30–40% of the total cost [11]. Thus, an effective pretreatment should be inexpensive and maximize accessibility to structural carbohydrates with restricted inhibitors production [12].

Physicochemical pretreatments such as dilute acid, sulphur dioxide, aqueous ammonia, steam explosion, liquid hot water, ammonia fiber explosion, alkaline or wet oxidation, ionic liquids, and microwave-assisted pretreatment [13,14] can promote high yields of fermentable sugars. Nonetheless, these approaches usually require high energy inputs, need corrosion-resistance, and, in some cases, high-pressure reactors. In addition, they are often limited by the lack of selectivity and produce undesired reaction products, which act as inhibitors for the subsequent microbial fermentation.

Biological pretreatments have received increased attention [5,9,12] due to low energy demand, low pollution generation, and simple procedures and equipment requirements. According to Zabed et al. [12], the delignification of lignocellulosic materials by fungal cultures or enzymatic pretreatments exhibit a similar performance. A bioprocess, such as fungal pretreatment of plant biomass [9], represents a low-cost and eco-friendly alternative to improve enzymatic hydrolysis and superior selectivity towards lignin and hemicellulose [15], avoiding the formation of inhibitors. White-rot fungi (WRF) have been widely investigated in pretreatments of agro-industrial residues [9] due to the effective delignification by the production of ligninolytic extracellular oxidative enzymes, including lignin peroxidase (LiP), manganese-dependent peroxidase (MnP), manganese-independent peroxidase (MnIP), and laccase (Lacc) [16]. However, some WRF also promote the hydrolysis of polysaccharides to provide carbon for fungal growth, which may cause a considerable loss of soluble sugars. Therefore, it is necessary to evaluate and select an appropriate fungal species-biomass type combination [17].

This study aimed to extract bioactive compounds from grape stalks, evaluate their antioxidant activities, and obtain fermentable sugars through hydrolysis of structural carbohydrates. In order to improve enzymatic saccharification rate and yield, the substrate was pretreated by solid state fermentation (SSF) using six WRF. Total polyphenolic compounds, fiber composition, fungal patterns of secreted enzymes, saccharification kinetics, and monosaccharides composition were also analyzed.

## 2. Materials and Methods

### 2.1. Grape Stalks and Fungal Strains

Grape stalks (*Vitis vinifera* L.) were obtained in a local winery “Adega Cooperativa de Alijó” (Douro region, Portugal) during the season 2016–2017. These residues were dried, ground with a 4 mm mesh, and stored at room temperature.

Six WRF strains deposited in the culture collection of the Biochemistry Laboratory at Universidade de Trás-os-Montes e Alto Douro (UTAD) were used: *Irpex lacteus* (Fr.) Fr. (UTAD 3), *Ganoderma resinaceum* Boud. (UTAD V20), *Bjerkandera adusta* (Willd.) P. Karst. (UTAD 100), *Trametes versicolor* (L.) Lloyd (UTAD 103), *Trametes* sp. (UTAD Tra), and *Phlebia rufa* (Pers.) M.P. Christ. (UTAD 156/UF206).

### 2.2. Fungal Solid State Fermentation of Grape Stalks

For pretreatment by SSF, two 10 mm agar plugs removed from the growing front of the potato dextrose agar plates of each WRF strain were used to inoculate sterilized (121 °C for 15 min) flasks containing 15.0 g of chopped grape stalks moistened with 45 mL of a previously described [18] minimal liquid medium (containing per litre: 5.0 g glucose, 1.0 g (NH_4_)_2_SO_4_, 1.0 g KH_2_PO_4_, 0.5 g MgSO_4_·7H_2_O, 0.1 g yeast extract, 0.1 g CaCl_2_·2H_2_O). Under static conditions, triplicate flasks for each fungus and abiotic controls were incubated at 27 °C and harvested after 21 days of SSF. Flask contents were suspended in 120 mL of deionized water and incubated on a rotary shaker at 100 rpm for 3 h. After solid/liquid separation, chilled extracts were filtered (Whatman GF/A), centrifuged, and aliquots were stored at −20 °C before determination of enzymes, antioxidant activities, and total polyphenols (TPP). Solid fractions were dried at 60 °C until constant weight, and were used for saccharification and biopolymers analysis.

### 2.3. Antioxidant Activities

Antioxidant activities were measured in the liquid extract obtained after SSF. Radical scavenging activity (RSA) on DPPH^•^ (2,2-diphenyl-1-picrylhydrazyl) was measured as described by Moreno et al. [19] using 22 µL of each sample in triplicate and 200 µL of DPPH^•^ solution in 120 µM methanol, incubated for 30 min in the dark and at room temperature. Absorbance at 517 nm was determined using a Biotek Powerwave XS2 plate reader (BioTek Instruments, Inc., Winooski, VT, USA) at 25 °C. The RSA on ABTS^+^ was measured as described by Ozgen et al. [20]. Briefly, a stock solution of 7 mM ABTS^+^ was diluted with 20 mM sodium acetate buffer (pH 4.5) to an absorbance of 0.70 (±0.02) at 734 nm. Then, 10 µL of triplicate samples were mixed with 190 µL of the radical solution and incubated for 6 min. Absorbance was determined at 25 °C using a referred plate reader. RSA was quantified by the formula:

(1) $$\mathrm{RSA}\;(\%)=\frac{\left(A_{\mathrm{Control}}-A_{\mathrm{Sample}}\right)}{A_{\mathrm{Control}}}\times 100$$.

Quantification of total antioxidant activity (TAC) was performed applying different concentrations of Trolox^®^ (6-hydroxy-2,5,7,8-tetramethylchroman-2-carboxylic acid) to obtain a standard curve [21]. Results were expressed as mg Trolox per gram of sample dry weight.

### 2.4. Determination of Total Polyphenols and Lignocellulose Composition

Total polyphenols (TPP) of aqueous fractions in untreated and fungal treated samples were quantified by the Folin–Ciocalteau method [22] at 750 nm, using gallic acid as the standard. TPP content was expressed as mg gallic acid equivalent (GAE) per gram of sample dry weight. To determine the dry matter content after aqueous extraction, residual fermented solids were dried to constant weight and ground (1 mm mesh, Retsch Zm 200, Haan, Germany). Dried samples were analyzed for total nitrogen as Kjeldahl N according to AOAC method nº 954.01 [23]. Briefly, samples were digested at 420 °C for 60 min. After cooling, digested samples were distilled in a Semi-automatic Kjeldahl Distillation Unit (UDK 139, VELP Scientifica, Usmate Velate, Italy) followed by titration with 0.1 N H_2_SO_4_. Crude protein (CP) was estimated multiplying the nitrogen percentage by the conversion factor (6.25). Neutral detergent fiber (NDF), acid detergent fiber (ADF), and acid detergent lignin (ADL) fractions were determined according to Robertson and Van Soest [24]. The concentration of hemicellulose (HC) was calculated as the difference between NDF and ADF, and cellulose (Cell) as the difference between ADF and ADL.

### 2.5. Enzyme Assays

Enzyme activities were determined at 25 °C using a Helios gamma UV–vis spectrophotometer. Ligninolytic activities were monitored according to Fernandes et al. [22] using 0.1 mL of crude enzymes extracts and the respective buffered substrate in a 1.5 mL total reaction volume. Briefly, the activities of Lacc and MnIP were measured at 420 nm (*ε*_420_ = 36 mM^−1^·cm^−1^) following the oxidation of 2.0 mM 2,2′-azino-bis(3-ethylbenzthiazoline-6-sulfonic acid) (ABTS; Sigma-Aldrich, Darmstadt, Germany) in 100 mM phosphate–citrate buffer pH 4.0, and in 100 mM tartrate buffer pH 5.0, respectively. To obtain MnIP activity, assays in the presence of 0.10 mM H_2_O_2_ were corrected by subtracting the laccase activity (assays at pH 5.0 without H_2_O_2_). MnP activity was determined in the presence of 0.10 mM MnSO_4_ in a 100 mM tartrate buffer pH 5.0 and 0.10 mM H_2_O_2_ by the formation of Mn^3+^-tartrate (*ε*_238_ = 6.5 mM^−1^·cm^−1^). LiP activity was determined in 100 mM tartrate buffer pH 3.0 by the formation of veratraldehyde (*ε*_310_ = 9.3 mM^−1^ cm^−1^) from 2.0 mM veratryl alcohol oxidation in the presence of 0.10 mM H_2_O_2_. Enzyme activity units are defined as l μmol of substrate oxidized per minute under the assay conditions. 

Carboxymethylcellulase (CMCase) and avicelase activities were measured according to IUPAC recommendations [25]. Briefly, hydrolysis was carried out in 50 mM citrate buffer pH 4.8 at 50 °C and 100 rpm, using 2% (*w*/*v*) carboxymethylcellulose and 1% (*w*/*v*) Avicel, during 30 min and 3 h, respectively. The reducing sugars released were determined by the 3,5-dinitrosalicylic acid (DNS) method [26], using glucose as a standard. Xylanase activity was determined under similar CMCase conditions, except that 1% (*w*/*v*) xylan solution was used as the substrate [18].

### 2.6. Scanning Electron Microscopy (SEM)

SEM in environmental mode (SEM/ESEM FEI QUANTA-400, Hillsboro, OR, USA) was used to evaluate fungal pretreatment on grape stalk samples, directly observed after being fixed with carbon double-sided tape on aluminum stubs. For the visualization, it was used a Low Vacuum Mode, with a partial pressure inside the chamber of 1.33 mbar and an acceleration voltage between 15–20 kV.

### 2.7. Enzymatic Hydrolysis of Holocellulose

Untreated and pretreated grape stalks were hydrolyzed in triplicate at 0.5% (*w*/*v*) consistency by a commercial enzyme complex Onozuka R-10 (Merck, Darmstadt, Germany) containing 40 U CMCase g^−1^, 80 U xilanase g^−1^, and 7 U Avicelase g^−1^ dry biomass in 50 mM citrate buffer pH 4.8 at 45 °C, and 100 rpm for 72 h in the presence of 0.01% sodium azide. The hydrolyzed material was centrifuged (12,000× *g* for 20 min), and soluble sugars were identified and determined. Controls were submitted to the same procedures.

### 2.8. Quantification of Soluble Sugars and HPLC Analysis

The amounts of total reducing sugars were measured by the DNS method [26]. Glucose, xylose, and galactose were also determined at 35 °C on a HPLC system (Gilson, Inc., Middleton, WI, USA) using a BioRad Aminex HPX-87H (BioRad, Hercules, CA, USA) column connected to a refractive index detector. Centrifuged samples were filtered through 0.2 μm filters and loaded (20 μL) using a Gilson 234 auto-injector. The flow rate of isocratic elution was set to 0.1 mL min^−1^ using a 5 mM H_2_SO_4_ dilution prepared in ultrapure water.

### 2.9. Data Processing and Statistical Analysis

All experimental points are the average values of at least three independent experiments. Average and standard deviation values were determined in a Microsoft Excel spreadsheet. One-way analysis of variance (one-way ANOVA) at a significance level of 5% was carried out with the STATISTIX 10 (Tallahassee, FL, USA) software to test the statistical significance of differences between the controls and fungal treatments. Thus, the null hypothesis was rejected when *p* < 0.05. To analyze the impact of WRF pretreatment, variables NDF, ADF, ADL, HC, Cell, and reducing sugars (RS) were evaluated through PCA, using the software SPSS Statistics 23 (IBM, Armonk, NY, USA).

## 3. Results and Discussion

### 3.1. Antioxidant Activities

**Figure 1:**
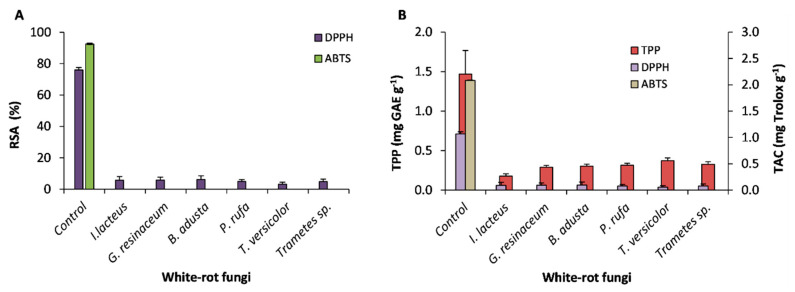
(**A**) Radical scavenging activity (RSA, %) of untreated and pretreated grape stalks determined with DPPH• and ABTS+ (means ± SD); (**B**) Quantitative analysis of total polyphenols (TPP) and total antioxidant activity (TAC) (means ± SD).

Bioactive compounds, such as antioxidants obtained from natural products, are a powerful tool in the food, chemical, and pharmaceutical industries in order to nullify the potentially harmful effects of free radicals in biological systems. The radical scavenging activity (RSA) of water-soluble extracts, from untreated and fungal pretreated grape stalks, were assessed by DPPH^•^ and ABTS^+^ assays, and are presented in Figure 1A. Untreated samples distinctly quenched DPPH^•^ (76.0% ± 3.0) and ABTS^+^ (92.5% ± 0.5). However, pretreated samples reach very low inhibition values, around 6% or zero, respectively, for DPPH^•^ or ABTS^+^. This loss of antioxidant activity is correlated with the observed fungal removal of phenolic compounds (Figure 1B). In fact, WRF are producers of phenolic-oxidizing enzymes and can also use several phenolic compounds as carbon sources [22], being both processes involved in phenolics removal.

Total antioxidant activity was quantified as Trolox equivalents, a water-soluble vitamin E analogue. The inhibition results with DPPH^•^ and ABTS^+^ showed that untreated grape stalks presented the highest antioxidant activity whose polyphenolic content was also the greatest among the samples considered (Figure 1B). Hence, a significant correlation (*p* < 0.05) between DPPH^•^ scavenging activity and TPP, expressed by the following equation: DPPH^•^ (% RSA) = 0.4737 × [TPP] (mg GAE L^−1^) − 12.291 (R^2^ = 0.9742), was found. According to Isah [27], different RSA may constitute further evidence on the relevance of the qualitative phenolic profile. Previously, it was reported that biological activity of grape stalks is associated with their polyphenolic composition [28,29] and their ability to scavenge free radicals.

### 3.2. Pretreatment: Lignocellulose Composition, Enzymatic Activities and PCA Analysis

Lignocellulosic substrates contain three main macromolecules: cellulose, an unbranched homopolysaccharide of glucose; hemicellulose, a heteropolysaccharide mainly containing 5-carbon and 6-carbon sugar monomers; and lignin, an amorphous phenylpropanoid heteropolymer. Lignocellulose structure depends on species, tissue, geographic origin, or even the growth period, which also seems to affect fungal behavior [30]. Given their recalcitrance, the deconstruction of the lignocellulosic matrix of grape stalks together with the high cost of enzymatic conversion are the main challenges to the efficient utilization and valorization of this residue. 

Samples of untreated and fungal-treated grape stalks were evaluated for contents of CP, NDF, ADF, ADL (Table 1), and TPP (Figure 1B). Crude protein is an important component as this substrate may be used as animal feed. No significant differences among samples were observed, which is in contrast with the results of Sousa et al. [31], who found an increase in protein content, but by using different plant wastes fermented by different fungal species. 

As presented in Table 1, the grape stalks contents of lignin, cellulose, and hemicellulose are in line [32] or higher [33] than those previously reported. Pretreatment plays an important role to overcome biomass recalcitrance, changing its structure and promoting the access to cellulose fibrils. Several fungal species can increase the bioconversion susceptibility of lignocellulosic substrates with variable effectiveness, depending on the substrate-fungus combination [9]. Since fungal degradation is a slow event [34], fungal screening of isolates with the highest enzyme activities plays an important role, despite preferential and selective lignin degradation also depending on the type of substrate [35].

**Figure 2:**
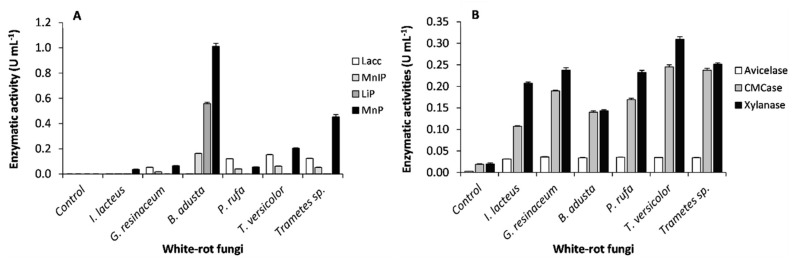
Enzymatic activities (means ± SD) measured at the end of the fungal solid state fermentation (SSF) of grape stalks. (**A**) Ligninolytic enzyme activities: Lacc, laccase; MnIP, manganese-independent peroxidase; LiP, Lignin peroxidase; MnP, manganese-dependent peroxidase; (**B**) Hydrolytic enzyme activities.

Seven enzymes (Figure 2) accounting for ligninolytic (LiP, MnP, MnIP, and Lacc) and hydrolytic activities (CMCase, xylanase, and avicelase) were evaluated at the end of SSF. Peroxidases (LiP, MnP, and MnIP) and Lacc were the major oxidative enzymes secreted by WRF responsible for lignin oxidation [9]. However, these enzymes are not always produced or detected in fungal cultures, probably due to the absence of an appropriate enzyme inductor, or the presence of inhibitors. 

As can be seen in Figure 2A, all six tested WRF produced MnP, which is the only ligninolytic activity detected in substrate pretreated by *I. lacteus*. Among peroxidases, the highest values of MnP (1.012 ± 0.023 U·mL^−1^), followed in decreasing order by LiP (0.558 ± 0.011 U·mL^−1^) and MnIP (0.162 ± 0.002 U·mL^−1^), were observed in grape stalks fermented by *B. adusta*, while no Lacc activity was detected. *P. rufa*, *T. versicolor*, and *Trametes* sp. show the same profile in terms of Lacc and MnIP production. Apart from MnIP, an identical profile was achieved in previous studies with wheat straw [18,36]. In the second best MnP producer, *Trametes* sp., this ligninolytic activity is approximately four times higher than laccase. In fact, higher levels up to approximately 10 times higher of the MnP activity than that of the laccase activity were also reported [37]. Although all fungi produced avicelase (Figure 2B), the observed average values were very low (0.034 U·mL^−1^) compared with CMCase and xylanase. For CMCase and xylanase enzymes, the most active producer was *T. versicolor* with a maximum value of 0.245 ± 0.005 U·mL^−1^ and 0.309 ± 0.006 U·mL^−1^, respectively. However, for *B. adusta*, xylanase activity was higher than CMCase. The production of lignocellulolytic enzymes during fungal SSF of agro-industrial crop residues has already been reported by several authors [18,36]. However, data regarding species *G. resinaceum*, *B. adusta*, and *P. rufa* are being presented for the first time using grape stalks as culture medium.

The main aim of enzyme production analysis was to identify, among the different WRF species, which one is better to improve cellulose/lignin ratio, i.e., increase delignification without extensive degradation of cellulose and hemicellulose. This previous pretreatment step plays an important role in the saccharification performance because it would increase the access of hydrolytic enzymes to structural polysaccharides [38]. However, as previously pointed out [36,39], the highest lignin degradation and MnP activity levels in fungal pretreated substrates were not necessarily correlated with the best soluble sugars yield obtained after saccharification. In this work, *G. resinaceum* was the best fungus to remove lignin, followed by *P. rufa*, *Trametes* sp. and *B. adusta*. Even though extracellular ligninolytic enzymes produced during pretreatment are the main ones responsible for lignin degradation, the presence or production of natural Lacc mediators and low molecular-weight oxidative agents [40] might also contribute to enhancing lignin biodegradation. 

**Figure 3:**
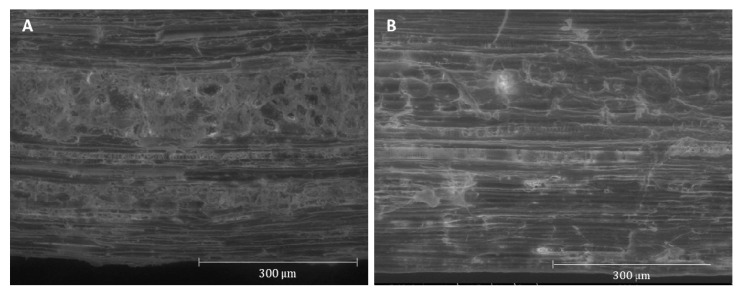
Scanning electron microscopy (SEM) photomicrographs of grape stalks samples: (**A**) untreated and (**B**) after *P. rufa* pretreatment.

The surface morphology of grape stalks before and after fungal pretreatment were visualized by SEM (Figure 3). It was observed that *I. lacteus* (results not shown) does not cause significant changes in substrate microstructure, a condition also reported by Salvachúa et al. [39]. However, samples pretreated by *P. rufa* (Figure 3B) revealed erosion of the lignocellulosic matrix. Less fine materials covered the cells, parenchyma cells were less compacted, xylemic vessels and phloem tubes with their internal ultra-structure were more exposed. In particular, typical xylemic inter-vessel walls arranged in a ladder-like series became more visible after SSF by *P. rufa*. These changes are consistent with the removal or modification of lignin and hemicellulose, making cellulose fibrils more exposed and accessible to the action of cellulolytic enzymes. In addition, partial but selective lignin barrier removal contributes to the observed increase of the cellulose/lignin ratio. A similar pretreatment effect was also observed with wheat straw [30,36] and corn stover [41].

**Figure 4:**
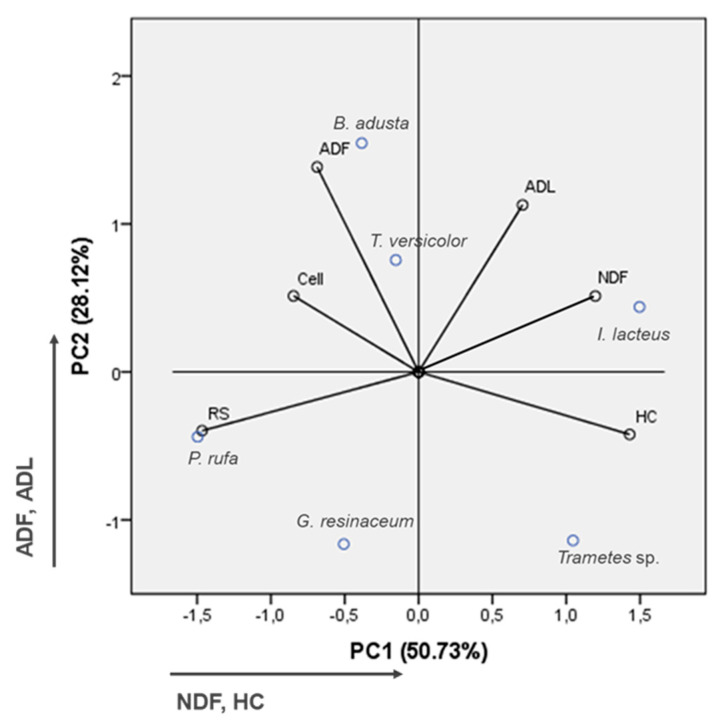
Principal component analysis (PCA) of fungal influence on the analyzed variables in pretreated grape stalks. NDF, ash-free neutral detergent fiber; ADF, ash-free acid detergent fiber; ADL, acid detergent lignin; Cell, cellulose; HC, hemicellulose.; RS, reducing sugars.

In order to better explain the observed variability, experimental data were submitted to a PCA analysis (Figure 4). Eigenvalues from PCA indicated that the first two principal components (PC) accounted for 78.85% of the total data variance (PC1: 50.73% and PC2: 28.12%). Axe PC1 was positively correlated to NDF and hemicellulose, and negatively with cellulose content and reducing sugars. Axe PC2 was positively correlated to ADF and ADL. In addition, PCA showed that pretreatment by *B. adusta*, *P. rufa*, and *G. resinaceum* is correlated with increased grape stalks delignification, and *T. versicolor* with lower lignin removal; *I. lacteus* and *Trametes* sp. are correlated with a high content in hemicellulose. Regarding reducing sugars, they were negatively correlated with NDF and ADL, while *P. rufa* was highly correlated with reducing sugars yield.

### 3.3. Enzymatic Saccharification

After the extraction of bioactive compounds and WRF pretreatment, grape stalks could be used for saccharification and subsequent conversion into value-added products such as biofuels, according to the biorefinery concept. Within the first two days of hydrolysis, enzymatic saccharification of pretreated grape stalks follows a pseudo first-order reaction kinetics, with estimated velocity constants (*k*) shown in Table 2. All fungal pretreatments made it possible to obtain significant (*p* < 0.05) increases in enzymatic saccharification when compared to non-inoculated controls (Table 2). However, sugar yields varied depending on fungal species used for preatment: *P. rufa* > *G. resinaceum* ≈ *T. versicolor* > *B. adusta* > *Trametes* sp. > *I. lacteus*.

Chemical and biological processes have been increasingly applied in two major fields: (i) the treatment of wastes and wastewaters pollution, and (ii) the pretreatment of lignocellulosic biomass for further bioconversion [42]. Among chemical processes, the so-called advanced oxidation process [43,44] has been used to eliminate recalcitrant organic contaminants, while dilute acid hydrolysis has been applied as a pretreatment of lignocellulosic materials [14]. Among biological processes, WRF incubations under submerged fermentation or SSF and enzymatic processes have gained increased attention both in the field of bioremediation and pollution abatement [22], as well as for biomass delignification [12,17,18]. However, fungal cultures usually exhibit long incubation times and greater or lesser carbohydrates loss as a function of fungal strain, while enzymatic pretreatments require costly processes of enzyme production, purification, and the preparation of enzymatic cocktails. In addition, enzyme inhibition by some natural-occurring phenolics [45], the stability of ligninolytic enzymes and consumption of H_2_O_2_ as a co-substrate for catalytic cycle of peroxidases, are limiting factors. Thus, WRF are an attractive option for biomass delignification and the deconstruction of the cell wall matrix, since this is an environment-friendly biotechnology and also a low-cost process.

Since, as we saw in Section 3.2, *P. rufa* is neither the best MnP producer nor the best delignifying fungus among the set of considered fungi, its best saccharification performance may lie in its ability to promote extensive removal of hydroxycinnamic acids [18]. This action, carried out through the hydrolysis of ester bonds, weakens cell wall integrity and helps further the enzymatic hydrolysis of structural polysaccharides because hydroxycinnamic acids, such as *p*-coumaric and ferulic acids, are bound to hemicellulose (e.g., xyloglucans) by ester bonds, and to lignin mainly by ether bonds. 

**Figure 5:**
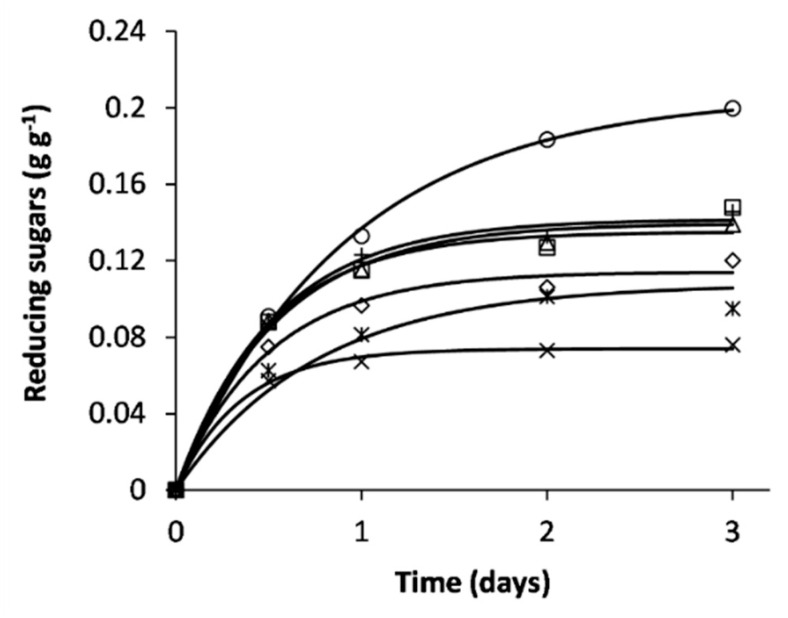
Kinetic profiles of enzymatic saccharification of grape stalks. Experimental points are means of triplicate experiments (SD < 2%): untreated grape stalks (×) and fungal pretreated grape stalks by *I. lacteus* (ж); *Trametes* sp. (◊); *B. adusta* (Δ); *T. versicolor* (+); *G. resinaceum* (□); *P. rufa* (○).

According to curve profiles in Figure 5, the reducing sugars production stabilizes over time, a pattern previously observed [46,47]. SSF by *P. rufa* proved to be the best fungal pretreatment. *P. rufa* significantly (*p* < 0.05) improves the enzymatic hydrolysis of structural polysaccharides, increasing the reducing sugars yield, pseudo first-order rate constant, and productivity, which is in line with reports obtained from other cellulosic substrates [36,41].

**Figure 6:**
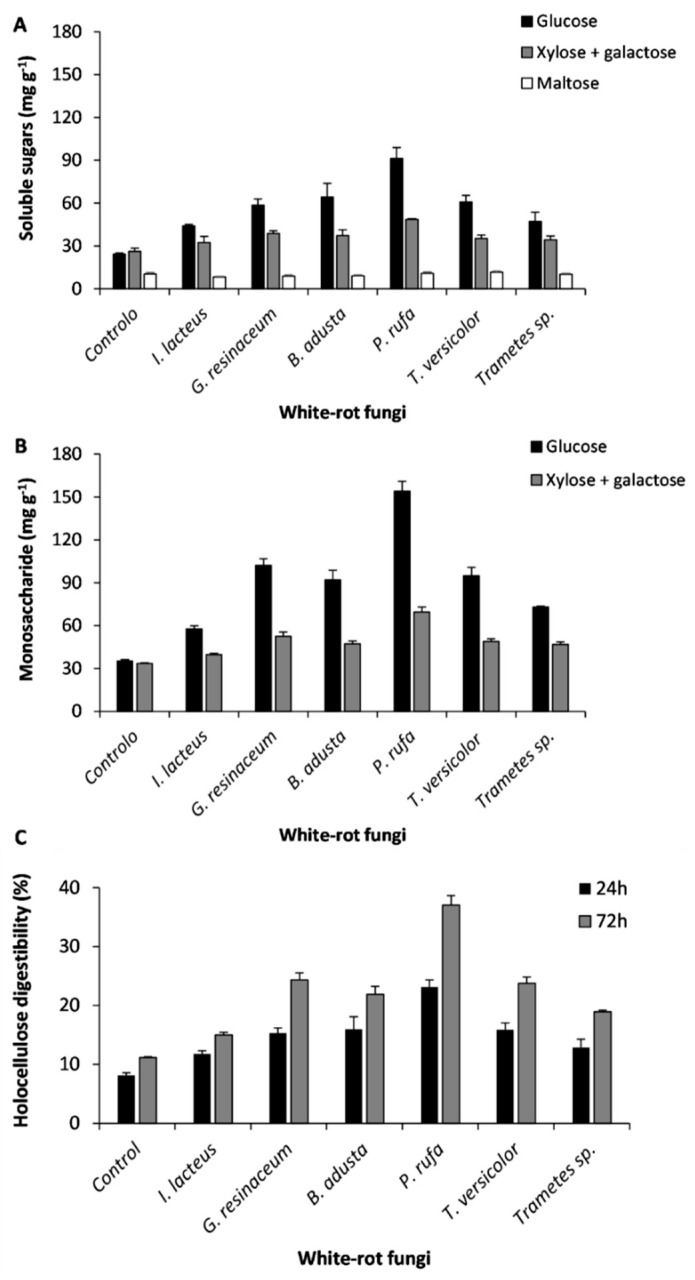
Reducing sugars recovered and quantified by high performance liquid chromatography (HPLC) after 24 h (**A**) and 72 h (**B**) of untreated and pretreated grape stalks hydrolysis; (**C**) Percentage of holocellulose hydrolysis.

The conversion of grape stalks into soluble sugars after 24 h and 72 h of hydrolysis is shown in Figure 6A,B. As can be seen, no disaccharides were detected after 72 h of hydrolysis, and the highest value of glucose production occurred in samples pretreated by *P. rufa* (154 mg glucose g^−1^ pretreated grape stalks) (Figure 6B). Concerning the monosaccharides ratio, it should be pointed out that the 1:1 ratio of glucose to the sum of xylose plus galactose, observed in untreated samples, changes to the value of 2:1 after pretreatment by *P. rufa*, *G. resinaceum*, *B. adusta*, and *T. versicolor*. After pretreatment with *P. rufa*, the hydrolysis of grape stalks holocellulose was 37% of the theoretical yield (Figure 6C), a remarkable conversion increase when compared to the other fungal pretreatments and untreated samples.

## 4. Conclusions

Grape stalks are a good source of bioactive compounds with antioxidant activity. However, they must be extracted before WRF pretreatment, due to the strong reduction of this compound’s category observed during SSF. A highly significant increase of the structural polysaccharides accessibility to enzymatic hydrolysis, up to three-fold, occurs after fungal pretreatment. Overall, chemical analyses complemented by SEM show that pretreatment with some WRF strains is able to promote erosion of plant cell walls. All fungal strains revealed the production of significant amounts of xylanase, CMCase, and ligninolytic activities, especially MnP. Also, enzymes MnP and Lacc seem to be important for successful biomass delignification. Pretreatment by *P. rufa* significantly increases monosaccharides production by enzymatic hydrolysis proving, for the first time, that *P. rufa* could be an effective fungal pretreatment of grape stalks. Saccharification obeys a pseudo first-order kinetics, and a nearly three-fold increase in velocity constant, yield, and productivity of reducing sugars was obtained. In addition, the ratio of glucose to the sum of xylose plus galactose increases from 1:1 to 2:1. Thus, after the extraction of bioactive compounds, *P. rufa* pretreated biomass can be hydrolyzed in order to obtain soluble sugars for further bioconversion into value-added products such as biofuels and other biomolecules. 

## Figures and Tables

**Table 1:** Chemical composition (% DM) of untreated and fungal pretreated grape stalks.

	CP	NDF	ADF	ADL	HC	Cellulose	ADL Removal (%)
Control	5.61	86.40 ^ab^	74.17 ^bc^	30.96 ^a^	12.23 ^ab^	43.21 ^e^	-
*I. lacteus*	4.65	88.17 ^a^	75.08 ^bc^	30.07 ^abc^	13.09 ^a^	45.01 ^bcd^	2.9
*G. resinaceum*	4.37	85.50 ^bc^	74.85 ^bc^	28.52 ^d^	10.65 ^abc^	46.34 ^ab^	7.9
*B. adusta*	5.11	87.02 ^ab^	77.48 ^a^	29.91 ^abc^	9.54 ^bc^	47.57 ^a^	3.4
*P. rufa*	5.29	83.73 ^c^	75.09 ^b^	29.56 ^cd^	8.64 ^c^	45.53 ^bc^	4.5
*T. versicolor*	5.29	85.11 ^bc^	75.21 ^b^	30.88 ^ab^	9.90 ^abc^	44.33 ^cde^	0.3
*Trametes* sp.	4.50	86.43 ^ab^	73.39 ^c^	29.67 ^bcd^	13.04 ^a^	43.72 ^de^	4.2
*p*-value	0.106	<0.001	<0.001	<0.001	0.001	<0.001	-

Values with different superscript letters (a, b, c, d, e) within each column are significantly (*p* < 0.05) different from each other. CP, crude protein; NDF, ash-free neutral detergent fiber; ADF, ash-free acid detergent fiber; ADL, acid detergent lignin; HC, hemicellulose.

**Table 2:** Parameters of enzymatic saccharification of untreated and fungal pretreated grape stalks.

	Sugars Yield (mg g^−1^)	*k*^(1)^ (d^−1^)	R^2^	Productivity (mg g^−1^ h^−1^)
Control	75.9 ± 0.4 ^f^	0.150 ± 0.010	0.867	1.0
*I. lacteus*	94.8 ± 1.0 ^e^	0.315 ± 0.006	0.943	1.3
*G. resinaceum*	147.7 ± 2.2 ^b^	0.224 ± 0.008	0.804	2.1
*B. adusta*	138.7 ± 1.0 ^c^	0.234 ± 0.004	0.844	1.9
*P. rufa*	199.6 ± 1.8 ^a^	0.447 ± 0.003	0.944	2.8
*T. versicolor*	145.5 ± 0.9 ^b^	0.254 ± 0.008	0.733	2.0
*Trametes* sp.	120.1 ± 0.6 ^d^	0.213 ± 0.002	0.812	1.7

^(1)^ Pseudo first-order rate constant determined during first two days of hydrolysis. Sugar yield: values with different superscript letters (a, b, c, d, e, f) are significantly (*p* < 0.05) different from each other.

## References

[1] Oh I., Yoo W.J., Yoo Y. (2019). Impact and Interactions of Policies for Mitigation of Air Pollutants and Greenhouse Gas Emissions in Korea. Int. J. Environ. Res. Public Health.

[2] Li Y., Chiu Y.H., Lin T.Y. (2019). Research on New and Traditional Energy Sources in OECD Countries. Int. J. Environ. Res. Public Health.

[3] Oh Y.K., Hwang K.R., Kim C., Kim J.R., Lee J.S. (2018). Recent developments and key barriers to advanced biofuels: A short review. Bioresour. Technol..

[4] International Renewable Energy Agency (IRENA) (2018). Renewable Energy Prospects for the European Union (REmap Analysis Conducted by the International Renewable Energy Agency in Co-Operation with the European Commission).

[5] Sindhu R., Binod P., Pandey A. (2016). Biological pretreatment of lignocellulosic biomass—An overview. Bioresour. Technol..

[6] Nicolini L., Volpe C., Pezzotti A., Carilli A. (1993). Changes in in-vitro digestibility of orange peels and distillery grape stalks after solid-state fermentation by higher fungi. Bioresour. Technol..

[7] Moldes D., Lorenzo M., Sanromán M.A. (2004). Different proportions of laccase isoenzymes produced by submerged cultures of *Trametes versicolor* grown on lignocellulosic wastes. Biotechnol. Lett..

[8] Levin L., Diorio L., Grassi E., Forchiassin F. (2012). Grape stalks as substrate for white rot fungi, lignocellulolytic enzyme production and dye decolorization. Rev. Argent. Microbiol..

[9] Wan C., Li Y. (2012). Fungal pretreatment of lignocellulosic biomass. Biotechnol. Adv..

[10] Isikgor F.H., Becer C.R. (2015). Lignocellulosic biomass: A sustainable platform for the production of bio-based chemicals and polymers. Polym. Chem..

[11] Bhutto A.W., Qureshi K., Harija K., Abro R., Abbas T., Bazmi A.A., Karim S., Yu G. (2017). Insight into progress in pre-treatment of lignocellulosic biomass. Energy.

[12] Zabed H.M., Akter S., Yun J., Zhang G., Awad F.N., Qi X., Sahu J.N. (2019). Recent advances in biological pretreatment of microalgae and lignocellulosic biomass for biofuel production. Renew. Sustain. Energy Rev..

[13] Den W., Sharma V.K., Lee M., Nadadur G., Varma R.S. (2018). Lignocellulosic biomass transformations via greener oxidative pretreatment processes: Access to energy and value-added chemicals. Front. Chem..

[14] Kumar M.N., Ravikumar R., Thenmozhi S., Kumar M.R., Shankar M.K. (2019). Choice of pretreatment technology for sustainable production of bioethanol from lignocellulosic biomass: Bottle necks and recommendations. Waste Biomass Valoriz..

[15] Baruah J., Nath B., Sharma R., Kumar S., Deka R., Baruah D., Kalita E. (2018). Recent trends in the pretreatment of lignocellulosic biomass for value-added products. Front. Energy Res..

[16] Ruiz-Dueñas F.J., Morales M., García E., Miki Y., Martínez M.J., Martínez A.T. (2009). Substrate oxidation sites in versatile peroxidase and other basidiomycete peroxidases. J. Exp. Bot..

[17] Su Y., Yu X., Sun Y., Wang G., Chen H., Chen G. (2018). Evaluation of screened lignin-degrading fungi for the biological pretreatment of corn stover. Sci. Rep..

[18] Dinis M.J., Bezerra R.M.F., Nunes F., Dias A.A., Guedes C.V., Ferreira L.M., Cone J.W., Marques G.S., Barros A.R., Rodrigues M.A. (2009). Modification of wheat straw lignin by solid state fermentation with white-rot fungi. Bioresour. Technol..

[19] Moreno S., Scheyer T., Romano C.S., Vojnov A.A. (2006). Antioxidant and antimicrobial activities of rosemary extracts linked to their polyphenol composition. Free Radic. Res..

[20] Ozgen M., Reese R.N., Tulio A.Z., Scheerens J.C., Miller A.R. (2006). Modified 2,2’-azino-bis-3-ethylbenzothiazoline-6-sulfonic acid (ABTS) method to measure antioxidant capacity of selected small fruits and comparison to ferric reducing antioxidant power (FRAP) and 2, 2’-diphenyl-1-picrylhydrazyl (DPPH) methods. J. Agric. Food Chem..

[21] Re R., Pellegrini N., Proteggente A., Pannala A., Yang M., Rice-Evans C. (1999). Antioxidant activity applying an improved ABTS radical cation decolorization assay. Free Radic. Biol. Med..

[22] Fernandes J.M.C., Sousa R.M.O., Fraga I., Sampaio A., Amaral C., Bezerra R.M.F., Dias A.A. (2020). Fungal biodegradation and multi-level toxicity assessment of vinasse from distillation of winemaking by-products. Chemosphere.

[23] Association of Official Analytical Chemists (1990). Official Methods of Analysis.

[24] Robertson J.B., Van Soest P.J., James W.P.T., Theander O. (1981). The detergent system of analysis and its application in human foods. The Analysis of Dietary Fiber in Food.

[25] Bezerra R.M.F., Dias A.A. (2004). Discrimination among eight modified Michaelis–Menten kinetics models of cellulose hydrolysis with a large range of substrate/enzyme ratios: Inhibition by cellobiose. Appl. Biochem. Biotechnol..

[26] Miller G.L. (1959). Use of dinitrosalicylic acid reagent for determination of reducing sugar. Anal. Chem..

[27] Isah T. (2019). Stress and defense responses in plant secondary metabolites production. Biol. Res..

[28] Anastasiadi M., Pratsinis H., Kletsas D., Skaltsounis A.-L., Haroutounian S.A. (2012). Grape stem extracts: Polyphenolic content and assessment of their in vitro antioxidant properties. LWT Food Sci. Technol..

[29] Barros A., Gironés-Vilaplana A., Teixeira A., Collado-González J., Moreno D.A., Gil-Izquierdo A., Rosa E., Domínguez-Perles R. (2014). Evaluation of grape (*Vitis vinifera* L.) stems from portuguese varieties as a resource of (poly) phenolic compounds: A comparative study. Food Res. Int..

[30] García-Torreiro M., López-Abelairas M., Lu-Chau T., Lema J. (2016). Fungal pretreatment of agricultural residues for bioethanol production. Ind. Crops Prod..

[31] Sousa D., Venâncio A., Belo I., Salgado J.M. (2018). Mediterranean agro-industrial wastes as valuable substrates for lignocellulolytic enzymes and protein production by solid-state fermentation. J. Sci. Food Agric..

[32] Ping L., Brosse N., Sannigrahi P., Ragauskas A. (2011). Evaluation of grape stalks as a bioresource. Ind. Crops Prod..

[33] Spigno G., Maggi L., Amendola D., Dragoni M., De Faveri D.M. (2013). Influence of cultivar on the lignocellulosic fractionation of grape stalks. Ind. Crops Prod..

[34] Yu H., Du W., Zhang J., Ma F., Zhang X., Zhong W. (2010). Fungal treatment of cornstalks enhances the delignification and xylan loss during mild alkaline pretreatment and enzymatic digestibility of glucan. Bioresour. Technol..

[35] Isroi, Millati R., Syamsiah S., Niklasson C., Cahyanto M.N., Ludquist K., Taherzadeh M.J. (2011). Biological pretreatment of lignocelluloses with white-rot fungi and its applications: A review. BioResources.

[36] Pinto P.A., Dias A.A., Fraga I., Marques G., Rodrigues M.A., Colaço J., Sampaio A., Bezerra R.M.F. (2012). Influence of ligninolytic enzymes on straw saccharification during fungal pretreatment. Bioresour. Technol..

[37] Hofrichter M., Vares T., Kalsi M., Galkin S., Scheibner K., Fritsche W., Hatakka A. (1999). Production of manganese peroxidase and organic acids and mineralization of ^14^C-labelled lignin (^14^C-DHP) during solid-state fermentation of wheat straw with the white rot fungus *Nematoloma Frowardii*. Appl. Environ. Microbiol..

[38] Pino M.S., Rodríguez-Jasso R.M., Michelin M., Flores-Gallegos A.C., Morales-Rodriguez R., Teixeira J.A., Ruiz H.A. (2018). Bioreactor design for enzymatic hydrolysis of biomass under the biorefinery concept. Chem. Eng. J..

[39] Salvachúa D., Prieto A., López-Abelairas M., Lu-Chau T., Martínez A.T., Martínez M.J. (2011). Fungal pretreatment: An alternative in second-generation ethanol from wheat straw. Bioresour. Technol..

[40] Janusz G., Pawlik A., Sulej J., Świderska-Burek U., Jarosz-Wilkołazka A., Paszczyński A. (2017). Lignin degradation: Microorganisms, enzymes involved, genomes analysis and evolution. FEMS Microbiol. Rev..

[41] Saha B.C., Qureshi N., Kennedy G.J., Cotta M.A. (2016). Biological pretreatment of corn stover with white-rot fungus for improved enzymatic hydrolysis. Int. Biodeterior. Biodegrad..

[42] Kumar B., Bhardwaj N., Agrawal K., Chaturvedi V., Verma P. (2020). Current perspective on pretreatment technologies using lignocellulosic biomass: An emerging biorefinery concept. Fuel Process. Technol..

[43] Rodríguez-Chueca J., Alonso E., Singh D.N. (2019). Photocatalytic mechanisms for peroxymonosulfate activation through the removal of methylene blue: A case study. Int. J. Environ. Res. Public Health.

[44] Lucas M.S., Dias A.A., Bezerra R.M., Peres J.A. (2008). Gallic acid photochemical oxidation as a model compound of winery wastewaters. J. Environ. Sci. Health Part A Tox. Hazard. Subst. Environ. Eng..

[45] Surendran A., Siddiqui Y., Saud H.M., Ali N.S., Manickam S. (2018). Inhibition and kinetic studies of cellulose and hemicellulose degrading enzymes of Ganoderma boninense by naturally occurring phenolic compounds. J. Appl. Microbiol..

[46] Gusakov V.A., Sinitsyn P.A., Manenkova A.J., Protas V.O. (1992). Enzymatic saccharification of industrial and agricultural lignocellulosic wastes—Main features of the process. Appl. Biochem. Biotechnol..

[47] Tian S.-Q., Ma S., Wang X.-W., Zhang Z.-N. (2013). Fractal kinetic analysis of the enzymatic saccharification of CO_2_ laser pretreated corn stover. Carbohydr. Polym..

